# Plant-Based Foods and Vascular Function: A Systematic Review of Dietary Intervention Trials in Older Subjects and Hypothesized Mechanisms of Action

**DOI:** 10.3390/nu14132615

**Published:** 2022-06-24

**Authors:** Massimiliano Tucci, Mirko Marino, Daniela Martini, Marisa Porrini, Patrizia Riso, Cristian Del Bo’

**Affiliations:** Department of Food, Environmental and Nutritional Sciences, Università degli Studi di Milano, Via Celoria 2, 20133 Milan, Italy; massimiliano.tucci@unimi.it (M.T.); marisa.porrini@unimi.it (M.P.); patrizia.riso@unimi.it (P.R.); cristian.delbo@unimi.it (C.D.B.)

**Keywords:** aging, vascular function, endothelial function, dietary intervention, intervention studies, systematic review, plant-based foods, vegetable foods, nitric oxide

## Abstract

Cardiovascular diseases, still the leading cause of mortality in the world, are closely related to vascular function. Older subjects are more susceptible to endothelial dysfunction and therefore it is important to define possible preventive or support strategies, such as consumption of foods with health-promoting effects. This systematic review aims to summarize the currently available evidence on acute or chronic trials testing the effect of selected plant-based foods on vascular function parameters in older subjects, and consider plausible mechanisms that may support the main findings. A total of 15 trials were included and analyzed, testing the effects of beetroot, plum, blueberry, and vegetable oils. We found some interesting results regarding markers of vascular reactivity, in particular for beetroot, while no effects were found for markers of arterial stiffness. The amelioration of vascular function seems to be more related to the restoration of a condition of nitric oxide impairment, exacerbated by diseases or hypoxic condition, rather than the enhancement of a physiological situation, as indicated by the limited effects on healthy older subjects or in control groups with young subjects. However, the overall set of selected studies is, in any case, rather limited and heterogeneous in terms of characteristics of the studies, indicating the need for additional high-quality intervention trials to better clarify the role of vegetable foods in restoring and/or improving vascular function in order to better elucidate the mechanisms through which these foods may exert their vascular health benefits in older subjects.

## 1. Introduction

According to the World Health Organization (WHO), cardiovascular diseases still represent the main cause of death in both developed and developing countries [1]. Cardiovascular diseases (CVDs) are thus unanimously recognized as a global burden, since they account for approximately one third of all deaths at global level [2]. The incidence of this chronic condition is still rising mainly in low-and middle-income countries, and can be attributable also to the worldwide increased life expectancy of population [2,3]. In particular, aging is associated with an increased risk for all CVDs, above all over 60 years [4,5,6,7]. In fact, it is estimated that 40% of the deaths in people aged >65 are caused by CVDs and their complications [8,9]. The mechanisms that lead to the increased cardiovascular (CV) risk during the aging process are related to both structural and functional changes in vascular systems [9,10].

Vascular endothelium represents the innermost monolayer of cells of the artery wall, directly in contact with blood flow, able to contribute to the regulation of vascular tone and hemostasis [11]. The endothelium exerts the aforementioned functions thanks to its capacity to perceive, through mechanical stimuli, changes in the blood flow and consequently induces smooth muscle cells of artery walls to relax [12,13,14]. One of the main vasodilator molecules involved in this process is nitric oxide (NO), a highly reactive soluble gas mainly synthetized by the endothelium through the enzyme nitric oxide synthase (NOS) from the amino acid arginine, which diffuses rapidly (half-life of 4–30 s) and acts on the surrounding cells in a paracrine fashion [15,16,17]. Thus, alterations of endothelial cell monolayer integrity represent a crucial factor in the onset of endothelial dysfunction and atherosclerosis, which is considered the underlying cause of most CVDs [18,19]. Atherosclerosis is a complex phenomenon that can be briefly described as a chronic, immunoinflammatory disease of large and medium arteries, fueled by lipids, and involving endothelial cells, leukocytes, and intimal smooth muscle cells [20]. The pathophysiology of atherosclerosis consists in the development of plaques formed by fibrous tissues and a lipid-rich core inside the tunica intima of artery walls [20,21,22]. These plaques can be unstable, and the acute rupture of these atheromatous plaques causes local thrombosis, thus leading to serious clinical consequences including stroke and heart disease [18,19]. Further metabolic dysregulations linked with endothelial dysfunction include insulin resistance, diabetes, and chronic kidney failure [23].

Aging is a gradual, continuous process of natural change that begins in early adulthood and that is associated with numerous cellular and molecular alterations such as genomic instability, telomere attrition, epigenetic alterations, loss of proteostasis, deregulated nutrient sensing, mitochondrial dysfunction, cellular senescence, and stem cell exhaustion [24]. Aging process is also characterized by vascular endothelial dysfunction [25,26]. This impairment affects all the major endothelium-derived vasodilators: NO, prostacyclin (PGI_2_), and the endothelium-derived hyperpolarizing factors (EDHFs); bringing to an increase of vasoconstrictor agents (e.g., endothelin-1, vasopressin, angiotensin II) [27]. Several mechanisms have been hypothesized; for instance, aging is recognized to promote an accumulation of reactive oxygen species at mitochondrial level such as superoxide (O_2_^−^) able to transform NO into peroxynitrite (ONOO^−^) and promote cumulative DNA damage. At endothelial level, the loss of NO and the consequent accumulation of high concentrations of ONOO^−^ induce the inactivation of mitochondrial manganese superoxide dismutase (the main enzyme involved in the hydrogen peroxide detoxification) and can switch the NO synthase via the oxidation of tetrahydrobiopterin from an NO- to an O_2_^−^- generating enzyme (NO synthase uncoupling) [28,29]. Both reactions lead to an increase in the concentration of O_2_^−^, in terms of a vicious circle, further promoting DNA damage, ONOO^−^ formation, and endothelial dysfunction. This alternation is also associated with a reduced capacity of endothelium to properly regulate vascular tone and exert antithrombotic activity, thus impacting negatively on tissue perfusion and allowing the onset of atherosclerosis [26,30,31].

According to the Global Burden of Disease Study 2019, diet-related risk factors represent the second risk factor for cardiovascular diseases mortality, after systolic hypertension, in both men and women [32]. Diets rich in fruits and vegetables are recommended for cardiovascular health; however, current global intakes of fruits and vegetables are well below recommendations (at least 5 portions per day) [33]. Extensive dose-response meta-analyses indicate significant cardiovascular risk reduction for high intake of fruit and vegetables [33,34,35,36,37]. For example, Wang et al. (2014) showed in four large prospective cohort studies that higher consumption of fruit and vegetables was associated with a reduction of risk of 4% for each additional serving per day of fruit and vegetables [35]. The large prospective cohort study of Miller and coauthors [36] found that the lower risk of non-cardiovascular events and total mortality was associated with high fruit, vegetable, and legume consumption (three to four servings per day, equivalent to 375–500 g/day). More recently, Zurbau and colleagues [37] reported in a meta-analysis of 117 observational studies (involving more than 4 million individuals and 100 thousand CV events) that total fruit and vegetables, fruit, and vegetables were associated with significant decreased incidence of cardiovascular disease (−7%, −9%, and −6% respectively) and associated mortality (−11%, −12%, and −13% respectively). This protective effect was particularly evident in older subjects. In fact, a greater adherence to a Mediterranean diet and/or more consumption of fruits and vegetables has been associated with a lower risk of arterial stiffness, vascular dysfunction, CV events, and mortality in older subjects [38,39,40] among other important functions [41,42,43,44]. The protective effects observed could be attributed to the large number of bioactive molecules contained in fruits and vegetables such as fibers, vitamins, minerals, phytochemicals (i.e., polyphenols, carotenoids), and essential fatty acids [45,46,47,48,49,50]. In fact, these compounds have shown several biological activities including favorable effects on endothelial function against ONOO^−^ formation and accumulation, but also through an inhibition of angiogenesis and cell migration and proliferation in blood vessels [51]. Given these premises and the urgent need to adopt preventive strategies in the context of healthy aging, the aim of this review is to systematically collect and summarize the main findings deriving from acute and chronic dietary intervention trials testing the health-promoting effects of plant-based foods (e.g., fruit, vegetables, nuts, legumes, cereal-based products, and vegetable oils) on markers of vascular function in older subjects. In addition, plausible mechanistic insights have been considered to support and explain the main positive findings.

## 2. Materials and Methods

### 2.1. Search Strategy

**Figure 1:**
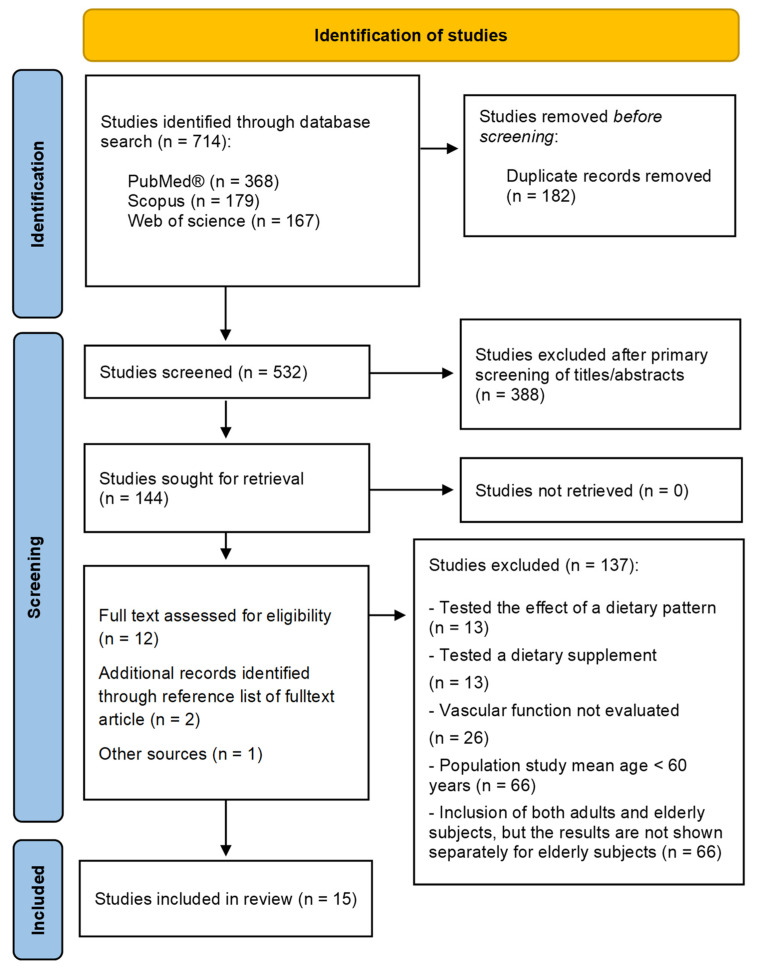
PRISMA flow chart of the systematic review literature search.

A systematic literature search was conducted using the three different academic digital databases PubMed^®^ [52], Web of Science [53], and Scopus [54]. The search was conducted in July 2021 and then updated at the end of January 2022. The following Boolean search strings were used, with proper adaptation for each database: “endothelial function OR vascular function OR reactive hyperemia OR EndoPAT) AND (aged OR elderly OR frail OR older) AND (vegetable* OR vegetable food* OR fruit* OR nut* OR legume* OR cereal*) AND (intervention OR trial OR clinical study)”. The search was limited to the last 10 years, from 2011 to 2021. To ensure completeness, the search was augmented by consulting the bibliographies of the eligible articles. The literature identification process, based on the PRISMA statement (Preferred Reporting Items for Systematic Reviews and Meta-Analyses) [55] is illustrated in Figure 1. The following systematic review was not recorded in any register and no review protocol was prepared.

### 2.2. Study Selection

Studies were considered eligible if they: (i) were published in the last ten years (from 2011 to 2021), (ii) were written in English, (iii) reported the results of a dietary intervention trial, (iv) tested the effect of a vegetable product, (v) analyzed markers of vascular/endothelial function, (vi) involved older subjects (population mean age >60 years; both sexes included).

Given these criteria, intervention studies were excluded when the effects of single compounds and/or supplements or specific dietary patterns (e.g., Mediterranean diet) were tested. Dietary intervention trials that involved subjects with different ages (from young to older subjects) were included only when data for older subjects were extractable.

### 2.3. Data Extraction and Presentation

Two authors (M.T. and M.M.) independently abstracted and tabulated data from eligible studies. Disagreement between reviewers was resolved through consultation with a third independent reviewer (D.M.) to reach a consensus. Details recorded included the year of publication, study design, location, sample size and characteristics of enrolled subjects, type and characteristics of tested plant food, characteristics of control, type of dietary assessment, primary and secondary outcomes, and results.

### 2.4. Risk of Bias

Risk of bias in individual studies and across the studies was carried out by two independent authors (D.M and C.D.B.). The evaluation was carried out following the criteria of the Cochrane Handbook for Systematic Reviews of Interventions. The analysis was structured into the following seven domains: (1) random sequence generation, (2) allocation concealment, (3) blinding of participants and personnel, (4) blinding of outcome assessment, (5) incomplete outcome data, (6) selective reporting, and (7) other bias. Each domain was judged as high risk, unclear risk, or low risk. Disagreements were resolved by consensus or seeking consultation with another author (P.R.).

## 3. Results

### 3.1. Study Selection

The study selection process is shown in Figure 1. A total of 531 records were identified from the database search (PubMed^®^, Scopus, and Web of science). After removing 120 duplicate articles, 411 studies were screened and 273 were excluded based on the title and abstract. The remaining 138 eligible papers were analyzed. At the of the process, 126 records were excluded for the following reasons: (i) mean population studied under the age of 60 years or data on older subjects not extractable, (ii) markers of vascular function not present, (iii) tested the effect of a dietary pattern (e.g., Mediterranean diet) or a single compounds supplement (e.g., grape polyphenols). At the end of the selection process, 12 trials were assessed for eligibility, and three additional trials were added following careful consultation of bibliography (two trials) and independent hand searching (one trial). Finally, 15 trials were included in this review; six studies evaluated the acute effect of vegetable foods while nine evaluated the chronic effects.

### 3.2. Study Characteristics

The main characteristic of the 15 studies included in the review are summarized in Table 1 [56,57,58,59,60,61] and Table 2 [62,63,64,65,66,67,68,69,70]. Specifically, Table 1 reports the results of acute interventions (*n* = 6), and Table 2 reports the results related to the chronic dietary interventions (*n* = 9). The majority of the studies (*n* = 7) were performed in the USA [57,58,59,60,62,67,68]. Of the remaining studies, four were conducted in the UK [61,65,66,69], two studies were performed in Australia [63,64], and two in Brazil [56,70]. The characteristics of the study population studied are heterogenous between the studies included in the review. Seven studies involved healthy subjects [57,58,59,61,62,66,69], four studies involved older subjects with cardiovascular risk factors [56,65,67,70], two studies involved subjects with overt peripheral artery disease [60,68], and one study involved subjects with mild cognitive impairment [63]. None of these studies involved only normoweight subjects. Eight studies included overweight subjects [57,58,59,62,63,66,68,69], while six studies included obese or both overweight and obese subjects [56,60,64,65,67,70]. One study included normoweight, overweight, and obese subjects [61]. Selected plant-based foods have been tested in relation to their possible effect on vascular function. The most studied food was the beetroot, mainly administered in the form of juice (from 70 mL up to 500 mL) [57,58,60,65,66,67,68,69], but also as powder to be resuspended in water (amount of powder *per se* not reported) [59,62] or gelatinized (100 g) [56]. Beetroot products were all analytically characterized for their content in nitrates. Other food sources included foods rich in anthocyanins (i.e., blueberry powder—30 g to be resuspended in 300 mL of semi-skimmed milk [61], and plum juice—250 mL/day [63,64]) and polyunsaturated fatty acids (i.e., different vegetable oils—30 mL/day) [70]. For the chronic studies, the length of the intervention ranged from 5 days [64] up to 90 days [70], while the acute study measured the investigated outcomes after at least 1 h [60], up to 3 h [58]. Regarding the assessment of vascular function, the main outcome considered was vascular reactivity (*n* = 8) [56,60,63,64,65,68,69,70], measured with different methods, but mainly through flow mediated dilation (FMD—*n* = 6) [56,60,64,65,68,69,70]. Additionally, arterial stiffness was frequently measured (*n* = 6) [56,59,60,66,68,70], mainly as an augmentation index (AIx—*n* = 4) [56,59,60,66].

Considering dietary and behavioral prescription, 6 acute studies included an overnight fast period before the evaluations. Four studies [57,58,59,61] requested the participants to refrain from exercise and alcohol and caffeine intake 24 h prior assessment. In the study of de Oliveira G. et al. [56], participants were also instructed to avoid the consumption of nitrates rich- food (e.g., beetroots, spinach, celery, kale, lettuce, red meats, sausages, and beans). In the study of Pekas et al. [60] volunteers were asked to maintain their habitual diet, but to track what they consumed the day before the first visit. In the study performed by using blueberries, participants were asked to avoid the consumption of foods and beverages rich in polyphenols such as fruit, vegetables, tea, and cocoa the day before the assessment [61].

### 3.3. Risk of Bias

In Supplementary Figures S1 and S2 are reported the risks of bias within individual studies and across the studies, respectively. Overall, the results have documented the selective reporting (reporting bias) and the blinding of outcome assessment (detection bias) to represent the highest risks of bias.

### 3.4. Results from Acute Intervention Studies

The results of short-term interventions are summarized in Table 1. Five studies were randomized and placebo-controlled [56,58,59,60,61] while one study [57] followed a parallel design comparing differences in vascular response between young and older subjects, without including a control group of older subjects. All studies were performed by using beetroot, while only 1 study was carried out by testing the effect of blueberry [61]. Three out of 5 studies administered beetroot in the form of juice [57,58,60], in dose ranging from 70 mL up to 500 mL, while 1 study used 100 g of beetroot gel [56], and 1 study a drink obtained by suspending beetroot powder (powder amount not reported) in 240 mL of water [59]. Beetroot administration provided from a minimum of 4 mmol [59] up to a maximum of 12.2 mmol of nitrates [56]. Apart from the comparative study of Hughes et al. [48], all studies that tested beetroot included a nitrate-deprived placebo in the same formulation as the test product. No information was available regarding the content of other bioactives contained in beetroot, such as betanin, which is known to be the main betalains of beetroot. Regarding blueberry, the tested product was a flavonoid-rich blueberry beverage (mainly providing anthocyanins i.e., malvidin, delphinidin, and petunidin; but also epicatechin oligomers) prepared by suspending 30 g of blueberry powder in 300 mL of semi-skimmed milk and by using a placebo drink containing the same amount and type of sugars and vitamin C of the test product [61].

Considering the outcomes, 2 studies evaluated vascular reactivity and arterial stiffness at rest [56,60], 2 study evaluated only arterial stiffness at rest [57,61], and the remaining 2 studies performed hemodynamics measures under during exercise [58,59]. In detail, de Oliveira G. et al. [56] found that administration of 100 g of beetroot gel resulted in a significant increase in FMD, reactive hyperaemia index (RHI) and blood flow velocity (BFV) compared to placebo, after 2 h from the administration. Additionally, Pekas et al. [60] found that administration of beetroot in the form of juice induced a significant increase in brachial and popliteal FMD, compared to placebo. Considering the arterial stiffness, none of the four acute studies found a significant effect on AIx and other related markers. In particular, Hughes et al. [57] compared the different response in AIx and AI@75 between healthy young and older adults after administration of 500 mL of a commercially available nitrate-rich beetroot juice beverage (providing 9.4 mmol/L of nitrates). The effect was evaluated at six different time points (baseline, 1 h, 1.5 h, 2 h, 2.5 h, and 3 h) post consumption of beetroot or a control product (no nitrate-depleted control product in older subjects). The results show that young adults presented significantly lower values of AIx at baseline compared to the older subjects. In addition, while AIx and AI@75 levels remained nearly constant along the time in the group of older subjects, a significant decrease in AIx and AI@75 was observed in the group of young adults. This effect was particularly greater at 2 h and 2.5 h.

Two out of six studies considered differences in vascular response during exercise after administration of beetroot. Hughes and colleagues [59] did not find any significant variation in hemodynamics parameters (i.e., blood flow—BF and vascular conductance—VC) measured at leg level at different intensity knee-extension exercise, after 2 h from beetroot juice ingestion in healthy older adult. Casey et al. [58] measured the difference in response between healthy young and older adults on hemodynamic measure (i.e., forearm blood flow—FBF, forearm vascular conductance—FVC) during exercise in both condition of normoxia and hypoxia. The authors found a significant increase after 3 h from consumption of beetroot juice, but not after placebo, of these parameters under hypoxia condition, compared to baseline values.

All studies performed on beetroot included the evaluation of nitrate and nitrite bioavailability. Four studies [57,58,59,60] evaluated the bioavailability at plasma or serum levels, while one study [56] considered nitrate and nitrate at urinary level. All studies reported a substantial increase of these compounds after beetroot ingestion while no significant effect was documented following placebo.

### 3.5. Results from Chronic Intervention Studies

Results from chronic intervention trials are summarized in Table 2. Six studies [62,65,66,67,68,69] tested the effect of beetroot, two studies were run evaluating the effects of plum [63,64], while one study used vegetable oils [70]. All studies were randomized and placebo-controlled. Half of them showed a parallel design, while the remaining half a crossover design. Considering the intervention, five out of six studies administered concentrated beetroot juice, ranging from 70 to 250 mL, and providing a minimum content of 4.2 mmol [62,68] up to 12 mmol of nitrates [66]. Only one study did not use beetroot juice, but supplied the participants with a drink obtained by suspending a concentrated beetroot powder (amount of powder not reported) providing 4 mmol of nitrates, to be resuspended in 200 mL of water [62]. In 5 out of 6 studies beetroot products had to be consumed daily, while in 1 study beetroot was provided three times/week (3 h prior to a training session) [68].

Considering the main outcomes investigated, most of the studies measured macro- and micro-vascular reactivity. Macrovascular reactivity was mainly evaluated through FMD [56,60] or different indexes such as ABI and RHBF [68]. Microvascular reactivity was assessed through numerous methods, but mainly measuring perfusion response after skin iontophoresis of ACh and SNP [65,69]. Only two studies evaluated the effect on arterial stiffness [66,67].

In detail, Gilchrist et al. [65] measured FMD and endothelium-independent dilation (response to 0.4 mg of sublingual nitroglycerine), as well as microvascular endothelial function evaluated through laser Doppler perfusion imaging in response to acetylcholine and sodium nitroprusside. The authors did not find any significant effect after two weeks of daily administration of 250 mL of beetroot juice (providing 7.5 mmol of nitrates) in a group of overweight/obese older subjects with T2DM. The same outcomes were evaluated by Jones and colleagues [69], that tested a lower portion of beetroot juice (70 mL, providing 4.7 mmol of nitrates) but for a longer period (4 weeks) in a group of non-obese older subjects. This study found an improvement in FMD after 2 and at 4 weeks compared to baseline, but not compared to placebo. Casey and Bock [62] evaluated the effect of 4 weeks consumption of 200 mL beetroot beverage (amount of powder beetroot not reported and providing about 4 mmol of nitrate) on different hemodynamic measures of shear profile at rest (derived from brachial artery diameter and mean blood velocity, both measured via Doppler ultrasound) and measure of exercise hyperemia during handgrip exercise (FBF—forearm blood flow, and FVC forearm vascular conductance). The authors found a significant improvement of pro-atherogenic shear stress measures and a lower pro-atherogenic oscillatory shear stress index in the beetroot group compared to the control group. Conversely, no effect on mean shear stress or anti-atherogenic antegrade velocity or antegrade shear stress was documented. The studies of Shaltout et al. [67] and Woessner et al. [68] involved an exercise intervention together with the consumption of beetroot juice. Shaltout et al. [67] used an impedance cardiograph to measure mean arterial pressure and obtained by derivation several hemodynamic measures (e.g., systemic vascular resistance, total arterial compliance, velocity index) after 6 weeks of aerobic training (3 times/week) associated with daily consumption of beetroot juice (70 mL, providing about 6 mmol of nitrates) or placebo juice (nitrate-deprived juice), in a group of 26 older subjects with controlled hypertension. However, the authors did not find any significant improvement in hemodynamic parameters, apart from the final value of total arterial compliance which was significantly increased compared to baseline, but in both the test and control groups compared to their baseline. Conversely, Woessner et al. [68] measured index of vascular function (i.e., resting ankle-brachial index—ABI, and reactive hyperemic blood flow—RHBF) after 12-week of 3 times/week mild training session associated with the consumption of 70 mL of beetroot juice (4.2 mmol of nitrates, to be consumed 3 h before each exercise training visit) or placebo in a group of 24 non-obese older subjects with a condition of peripheral artery disease associated with intermittent claudication. The study found a significant improvement in ABI and RHBF parameters compared to baseline in the group of subjects consuming beetroot and performing exercise, but not in those consuming the placebo. Finally, the study of Oggioni et al. [66] focused on arterial stiffness, but did not find any significant improvement in AIx after one week of daily consumption of two portions of 70 mL of beetroot juice (providing a total amount of 12 mmol of nitrates) in 20 healthy, non-obese older subjects.

Two studies tested the effect of a medium-long term intervention with a daily consumption of 250 mL anthocyanins-rich plum juice (*Prunus salicina* cv. Queen Garnet; providing about 200 mg of total anthocyanins, mainly constituted by cyanidin-3-rutinoside and cyanidin-3-glucoside). Both studies were conducted in Australia by the same research group [63,64]. In one study, 16 overweight healthy older subjects consumed 250 mL of plum juice per 4 days at the end of which an acute evaluation was performed. Within this test, a high energy/high fat meal (providing approximately 850 kcal and 65 g of total fats) was accompanied by the intake of 250 mL of a high anthocyanins plum juice or placebo [64]. The intervention documented a significant improvement in FMD, as well as post-occlusive reactive hyperemia (PORH), in the group consuming plum juice compared to control. In the second study the same authors tested the effect of 250 mL of two different plum juices (presenting low and high anthocyanins content, equal to 45 and 200 mg respectively) or placebo (apricot juice) on the vascular reactivity of 31 obese older subjects with mild cognitive impairment [63]. The study, a randomized, parallel design, involved a period of 8-week intervention in which participants consumed 250 mL/day of plum or a placebo juice. At the end of the intervention period, no significant differences in vascular reactivity were detected.

Finally, one study tested the effects of a daily consumption of 30 mL of three different vegetable oils (i.e., olive, flaxseed, and sunflower oil), providing mainly monounsaturated and polyunsaturated fatty acids, on FMD and carotid intima-media thickness—CIMT in a group of overweight elderly subjects [70]. This study followed a randomized, uncontrolled, parallel design and it was 90-day long. At the end of the trial, a significant improvement in CIMT was observed after vegetable oils supplementation. In addition, a significant improvement in FMD was also documented but only in the group of subjects consuming sunflower oil.

### 3.6. Other Markers Analyzed and Main Findings Obtained from Acute and Chronic Intervention Studies

Other markers, linked to cardiovascular risk, but not specifically related to vascular function, were considered within the studies included. Here, we have reported a brief report of the results obtained for vegetable products.

Regarding beetroot, several studies involved the assessment of blood pressure markers (e.g., systolic blood pressure, diastolic blood pressure, mean arterial pressure, pulse pressure), measured at brachial or aortic level, but most of them did not find any significant variation [56,58,59,62,65,66]. However, three studies [60,67,69] found a reduction in systolic blood pressure [60,67], while one study [69] reported a decrease in both systolic and diastolic blood pressure. Furthermore, Oggioni et al. [66] also considered the effect of beetroot in cardiac output at rest and during cardiopulmonary exercise testing, but without finding a significant effect. Shaltout et al. [67] tested the effect of beetroot on peak oxygen consumption and time to exhaustion during aerobic exercise in their study on hypertensive subjects, without showing a significant improvement. Pekas et al. [60] found a beneficial effect of beetroot in maximal walking time and levels of deoxygenated hemoglobin in older subjects with peripheral artery disease, while Woessner et al. [68] reported a significant effect on claudication onset time and tissue deoxygenation characteristics in subjects with peripheral artery disease with intermittent claudication.

Considering biochemical parameters, the effects were tested on biomarkers of inflammation (i.e., interleukin 6, IL-6), as well as endothelial integrity (e.g., E-Selectin, P-Selectin), and glucose metabolism markers (including insulin sensitivity), but without finding any significant effect [65,66].

Regarding anthocyanin (ACN)-rich foods (plum and blueberry), the two chronic studies also evaluated blood pressure and several metabolic and functional parameters associated with vascular health such as lipid profile and inflammation showing different results. One study [64] reported a significant effect of plum juice in the reduction of C-reactive protein (CRP) levels and a trend for the reduction of IL-6, but no effect on blood pressure, triacylglycerol, and total cholesterol, while the second study [63] showed a significant reduction in the levels of tumor necrosis factor (TNF)-α, but not on IL-6, IL-1β, CRP and blood pressure. Regarding blueberries [61], no significant effect was found for cognitive function or levels of brain-derived neurotrophic factor, but there was a trend for systolic blood pressure, which increased less in the blueberry group compared to controls. Finally, the study performed by using vegetable oils [70] evaluated also the impact on biochemical parameters such as CRP, apolipoprotein (Apo) B, Apo A, and ApoB/Apo/A ratio, but unfortunately no significant effect was reported.

## 4. Discussion

The aim of this review was to assess the evidence deriving from intervention trials testing the vasoactivity of bioactive-rich vegetable foods in older subjects. Overall, the number of trials is limited and involve mainly the beetroot. The discussion has been organized in different sections, discussing the main results in relation to each plant-based food and by providing the potential mechanisms identified from preclinical models and, when available, theorized by the authors of the papers included in the present revision.

### 4.1. Beetroot

Considering the effects of acute beetroot intake on vascular reactivity, two studies indicate an increase in FMD and related markers. The effects were observed in subjects with CV factors or peripheral artery disease (PAD) [56,60], suggesting a potential short-term contribution of beetroot in the modulation of FMD, RHI and BFV in subjects at risk. Regarding chronic studies, the majority of dietary interventions reported no effect following beetroot consumption. For example, Gilchrist et al. [65] did not find any significant effect on FMD and microvascular endothelial function after 2 weeks of daily administration of 250 mL of beetroot juice (providing 7.5 mmol of nitrates) in a group of overweight/obese older subjects with T2DM. Oggioni et al. [66] reported no variation on arterial stiffness parameters (e.g., AIx) after 1-week intervention with 70 mL of beetroot juice, provided twice a day (12 mmol nitrate), in healthy older subjects. Similar findings were also found by Jones and colleagues [69] showing a lack of significance on hemodynamic measures after 4-week intervention with a comparable dose of beetroot juice (70 mL providing 4.7 mmol of nitrate) in a group of healthy older subjects. This discrepancy could be attributed to the different population considered; in fact, while acute studies were conducted in subjects with CV factors, chronic interventions were carried out mainly on healthy subjects. Another plausible reason could be related to the rapid metabolization of nitrites that are converted into NO and quickly disappear from blood [17]. Finally, it is considered quite unlikely an increase in NO levels above a certain threshold in healthy subjects through NOS-independent mechanisms, given that in healthy subjects the impairment in NO production is less pronounced than in subjects who already show an endothelial dysfunction. However, some chronic studies have reported a beneficial effect on vascular function following the consumption of beetroot but in non-older subjects. For example, Asgary and co-workers [71] found an increase in FMD after 2 weeks in a group of 24 subjects presenting mild hypertension (systolic blood pressure between 130–140 mmHg), while Velmurugan et al. [72] documented an improvement after 6-week beetroot consumption in subjects with CV factors.

The rest of the studies were carried out in older subjects where the effect of beetroot was combined with physical activity. Casey et al. 2015 [58] indicated a significant effect of acute beetroot juice intake (500 mL providing 9.4 mmol of nitrate) on FBF and FVC in subjects performing physical exercise in a hypoxia condition (80% arterial O_2_ saturation, induced by a tight-fitting oronasal facemask), while no improvement was found when tested in normal conditions (normoxia) or in young subjects. Hughes et al. 2016 and 2020 documented a lack of significant effect on several vascular and hemodynamic markers (e.g., leg blood flow, vascular conductance AI, AI@75) following the acute intake of beetroot beverage (240 and 500 mL) measured during physical exercise and at different times in normoxia [57,59]. When considering chronic intervention, Casey and Bock [62] found that 4-week intervention with beetroot (providing at about 0.4 mmol of nitrate and 0.3 mmol of nitrite) improved conduit artery shear profiles, thus suggesting a reduction in the resistance of the arteries and a restoration of normal laminar flow in older adults. This result was not observed in a condition of handgrip exercise hyperemia. Woessner and coworkers [68] documented a significant increase in vascular parameters (ABI and RHBF) following 12-week intervention with 70 mL beetroot in subjects with PAD and intermittent claudication. The analysis of vascular parameters was carried out before and after a rehabilitation process that included 30 min of walking 3-time per week. Conversely, Shaltout and co-workers [67] showed no significant effect on hemodynamic markers in a small group of hypertensive older subjects after 6 weeks intervention (70 mL of beetroot per day, providing 7.5 mmol nitrate) associated with an aerobic exercise training (three times per week). The lack of univocal findings could be attributed to numerous factors. First, the different characteristics of the study population considered within the studies (e.g., healthy subjects, subjects with risk factors); second, the different exercise training program attended by the subjects during the experimental period that cannot be comparable; third, the difference in terms of experimental design including the amount of beetroot administered, nitrate content, duration of intervention, presence/absence of the control group and its characteristics; fourth, differences in terms of biomarkers analyzed that make the comparison difficult.

In respect to the effects on physical exercise, some premises are required. During the activity in normoxia, local vasodilation responds to the increased needs of skeletal muscles of oxygen for the oxidative phosphorylation of mitochondria, nutrients, as well as for the need to remove metabolites and dissipate the excess heat produced [73]. In order to respond adequately to these requests, the mechanisms of local vasodilation need to be multiple and involve not only vasoactive endothelial molecules (e.g., NO and PGI2), but also other factors (e.g., K^+^ and adenosine produced directly by the muscles under stress, as well as mechanical effect of the “muscle pump”) [74]. In fact, exercise hyperemia can also occur when NO and PGI2 are inhibited and it is estimated that NO contributes to exercise hyperemia only about 20% [75]. Thus, NO impairment during physical exercise is less easy to evaluate. However, exercise during a hypoxic condition depends much more on NO, probably since hypoxia induces further vascular adaptations to cope with the reduction of oxygen supply [76]. In fact, trials that evaluated exercise hyperemia during hypoxic conditions show that the consumption of beetroot could compensate for the lack of NO production in case of impairment by providing nitrate and nitrite to use for its manufacture through alternative ways than NOS, as reported above.

These data support the idea that when NO levels are not impaired (as occurs in healthy subjects) there is no beet effect, but also that the deoxygenation of red blood cells can induce an increase in their ability to release nitric oxide from nitrate and nitrites, as suggested in the Dinenno review [75]. These results seem also in line with the observations reported in the review of Jones [77], which indicates that the ergogenic effect of beetroot seems more easily detectable for activities shorter than 30 min, that generally are more intense than longer activities and thus more likely to recruit the contribution of the endothelial mechanism of vasodilation.

**Figure 2:**
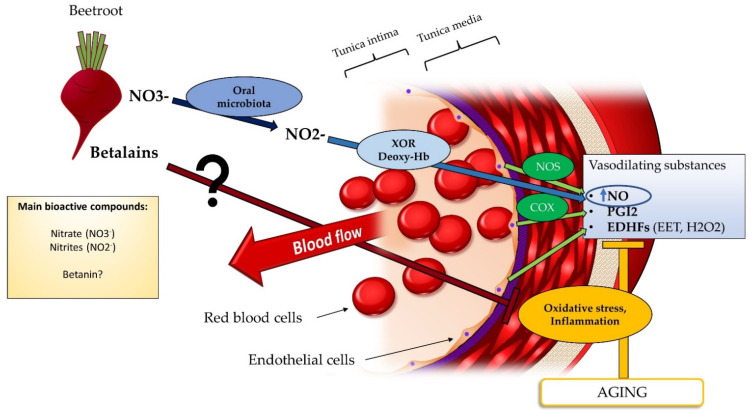
Schematic representation of the mechanism of action of beetroot in the increase of vascular function in older subjects. Endothelial cells produce vasodilating substances through different enzymes in response to physiological stimuli. Aging can be associated with high levels of oxidative stress and inflammation that cause an impairment of those vasodilating substances. Dietary NO3^−^ from nitrates rich vegetables, such as beetroot, can acutely contribute to replenish the pool of NO in case of impairment thanks to NOS independent pathway, such as XOR and Deoxy-Hb of red blood cells. Other beet bioactives (e.g., betanin, the main betalains contained in beetroot) can also be involved in these processes, through synergistic or independent effects. Legend: COX, cyclooxygenase Deoxy-Hb, deoxyhemoglobin; EDHFs, endothelial derived hyperpolarizing factors; EET, epoxyeicosatrienoic acid; H_2_O_2_, hydrogen peroxide; NO2^−^, nitrites; NO3^−^, nitrate; NO, nitric oxide; NOS, nitric oxide synthase; PGI2, prostacyclin2; XOR, xanthine oxidoreductase.

In Figure 2 are summarized the main potential mechanisms through which nitrates (NO3^−^), nitrite (NO2^−^) could exert a vasodilatory effect, perhaps in synergy with other beetroot compounds such as vitamins, minerals and betalains (e.g., betanin, isobetanin, vulgaxanthin I); these latter are responsible for the red color of the root [78]. Several studies have reported a health-promoting effect of betalains [79], thus their potential contribution in the modulation of vascular function should not be excluded. Accumulating evidence indicates that NO3^−^ and NO2^−^ can be reduced to NO, that can thus be obtained independently from arginine transformation catalyzed by NOS. The conversion of NO3^−^ into NO is dependent on the activity of the oral microbiota that is able to reduce NO3^−^ into NO2^−^. The latter are then subsequently reduced into NO thanks to the activity of the enzyme xanthine oxidoreductase (XOR) and deoxyhemoglobin (Deoxy-Hb) at the level of red blood cells [80,81]. In this regard, Ghosh et al. [82] documented that the beneficial effect on blood pressure after beetroot consumption was due to the conversion of NO3^−^ into NO by XOR, and that this conversion occurred significantly more in hypertensive subjects compared to normotensive controls.

Regarding safety issues, the European Food Safety Authority (EFSA) has set the Acceptable Daily Intake (ADI) for nitrate at 3.7 mg/kg (approximately 260 mg for a 70 kg adult, equivalent to 4,2 mmol). A similar limit is also suggested by the World Health Organization (WHO) [83]. Safety limits exist since the intake of nitrites can brings to the formation of carcinogenic compounds (i.e., N-nitrous compounds) or to the formation of methaemoglobin, which is particularly dangerous for infants [84]. It should be taken into account that the majority of the studies testing beetroot products included in this review used a dosage higher than EFSA’s ADI [56,57,58,60,65,66,67].

### 4.2. Effect of Fruit on Vascular Function

The role of fruits in the modulation of vascular function has been debated for a long period. Fruit is a rich source of numerous bioactive compounds such as vitamins, minerals, and phytochemicals including (poly)phenols and carotenoids. Three studies investigated the effects of fruit on vascular health in older subjects. Two were chronic studies testing the effects of plum [63,64], while one was an acute study carried out with blueberries [61].

Regarding the effect of plums, a study has shown to counterbalance the detrimental effect a high-calorie and high-fat meal over a period of four days in a group of overweight/obese older subjects [64]. Conversely, another study showed no effect on micro-vascular reactivity at the end of an 8-week intervention with plum in older subjects with mild cognitive impairment [63]. However, the authors were able to document a reduction in TNF-α serum levels, suggesting a contribution of the intervention on the inflammatory response. These two studies are difficult to compare due to their heterogeneity in terms of study population, duration and experimental design adopted. Another consideration is related to the dietary prescription followed by the subjects within the intervention periods. In the first study [64], participants were requested to avoid ACN-rich foods and a standardized, low-flavonoid, frozen meal was provided at dinner prior to the experiment. In the second study [63], subjects maintained their habitual diet. It cannot be excluded that this lack of standardization could have affected the results on vascular function.

To the best of our knowledge, only one study investigated the effects of blueberry in older subjects. Specifically, Dodd et al. [61] evaluated the acute effect of a highbush blueberry beverage (30 g of blueberry powder providing at about 580 mg of ACNs, mainly constituted by malvidin, delphinidin, and petunidin, while Queen Garnet plum result as mainly rich in cyanidin) on arterial stiffness in a group of 18 healthy older subjects. The results have documented a lack of significant effect. These findings seem in line with the observations deriving from acute blueberry studies, in which a significant improvement on vascular function, but not arterial stiffness, was documented in young/adult healthy subjects [85,86,87,88,89]. Additionally, the results from chronic intervention studies failed to observe an effect on arterial stiffness from the consumption of blueberry [90]. Conversely, Johnson and colleagues [91] found an amelioration in arterial stiffness in post-menopausal women following 8 weeks intervention with a drink containing 22 g of a highbush blueberry powder. Specifically, the authors documented a significant decrease in blood pressure and brachial-ankle pulse wave velocity (marker of arterial stiffness), but not in carotid-femoral pulse wave velocity. The authors attributed the beneficial effects to an increase in NO levels than to a reactive oxygen species (ROS) reduction, since the levels of superoxide dismutase (SOD) did not appear to be increased.

**Figure 3:**
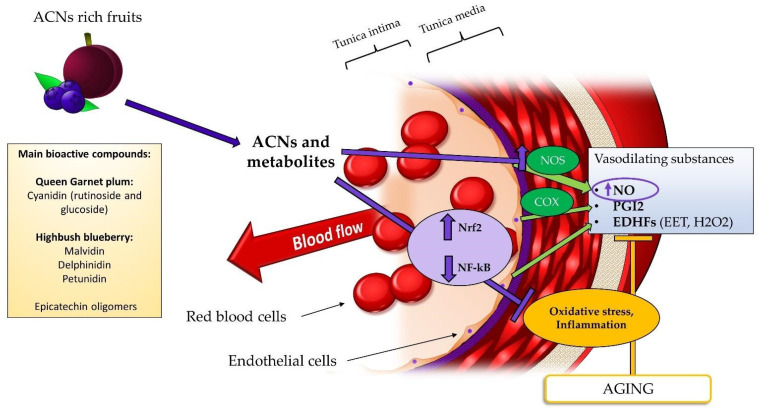
Schematic representation of the mechanism of action supposed for ACNs rich fruits in the increase of vascular function in the elderly. In contrast to nitrates that act by increasing NO levels through NOS-independent pathways, ACNs increase the expression of NOS and counteract the detrimental effects of oxidative stress (due to activation of Nrf2, that lead to increased defense from ROS) and inflammation (inhibiting the activation of NF-kB), thus increasing NO production and contribute to the restoration of its availability. Legend: ACNs, anthocyanins; COX, cyclooxygenase; EDHFs, endothelial derived hyperpolarizing factors EET, epoxyeicosatrienoic acid; H_2_O_2_, hydrogen peroxide; NF-kB, nuclear factor kappa B; NO, nitric oxide; NOS, nitric oxide synthase; Nrf2, nuclear receptor factor 2; PGI2, prostacyclin 2.

The putative mechanism of actions through which these ACNs could have a potential health benefit on vascular function is depicted in Figure 3. ACNs are the most studied bioactives in the CV field in relation to numerous experimental evidence that indicate a health-promoting effect, deriving from both in vivo studies (observational and randomized controlled trials, RCT) and in vitro studies [92,93]. Experimental evidence indicates that ACNs are capable of positively affecting vascular function, inflammation, and counteract pro-atherogenic processes [94]. Consistent literature indicates that these beneficial effects are mainly mediated by a regulatory effect on numerous genes involved in endothelial function especially by inducing an increased NO production [46,95,96,97]. This effect seems to be mediated by an increased activity of eNOS and/or by its preservation from the oxidation. Moreover, ACNs are considered as antioxidant compounds thanks to their capacity to counteract the detrimental effect of ROS by preventing the formation of ONOO^−^ and consequently the reduction of NO availability [98]. Several reviews have tried to explain the antioxidant activity of ACNs. One putative mechanism is related to their direct antioxidant activity against ROS, although this effect is influenced by the actual bioavailability [99]. The second more plausible mechanism consists in the regulation of genes involved with oxidative stress, such as antioxidant defense enzymes (e.g., SOD, catalase (CAT), and glutathione peroxidase (GPx)), through the activation of nuclear receptor factor 2 (Nrf2). In this regard, Rodriguez-Mateos et al. [89] found that the increase in vascular function observed in their study corresponded to a significant reduction in nicotinamide adenine dinucleotide phosphate (NADPH) oxidase activity, an enzyme that catalyzes the formation of ROS [91]. ACNs are also able to inhibit the activation of nuclear factor-kappa B (NF-Kb), thus interfering with inflammation response also at endothelial level by reducing the production of several interleukins and cytokines with a direct effect on vascular function [93,96].

### 4.3. Effect of Vegetable Oils on Vascular Function

The impact of vegetable oils on vascular function in older subjects has been evaluated only in one study [70]. de Oliveira P. et al. [70] performed a 12-week intervention testing the effect of 30 g/day of three different vegetable oils (flaxseed, olive, and sunflower oil) on markers of vascular function (FMD) and stiffness (carotid intima-media thickness (CIMT)). The results have shown a different effect that was dependent on the type of oil. Specifically, a significant amelioration of vascular response (evaluated by FMD) was reported only in the group that consumed sunflower oil, while a significant reduction in CIMT was documented in all the three intervention groups, even if the effect seemed to be more pronounced in the group of subjects consuming flaxseed and olive oils [70]. These results could be explained by the different composition in fatty acids (FAs); sunflower is rich in ω-6 PUFA (linoleic acid—LA), flaxseed oil in ω-3 PUFA (α-linolenic acid) while olive oil is rich in MUFA (oleic acid). Although the role of FA on vascular function has been poorly investigated and results from in vivo studies are controversial [100], growing evidence from preclinical models seems to suggest a relevant influence of FA in this context in both positive and negative ways. In particular, FA can be associated with vascular function through the modulation of inflammatory processes, oxidative stress, apoptosis of endothelial cells, and NO production [101,102]. In fact, ω-3, -6, and -9 FA differently modulate processes associated with vascular function and CV risk. Results from both in vivo and in vitro studies indicate that ω-3 could have a beneficial effect on vascular endothelium [103]. Among them, it has been observed that ω-3 is capable of increasing the availability of NO in the aorta of rats through the activation of eNOS and the enhancement of NOS activity [104]. Moreover, NO increase, induced by ω-3, can prevent the formation of oxidized-LDL (ox-LDL) due to the antioxidant effect of NO via the termination of radical chain propagation reactions [105]. In addition, evidence from an apoE-knockout mice model suggests that ω-3 play an important role on oxidative stress by increasing the activity of antioxidant enzymes (i.e., SOD, CAT, and GPx), determining lower levels of ROS [106]. Since oxidative stress induces endothelial cell damages, inflammation, cell permeability, increased apoptosis, and vascular remodeling [107], the capacity of ω-3 to counteract the excess of free radicals could represent a relevant mechanism in preventing vascular dysfunction. Beside antioxidant properties, ω-3 are also involved in the reduction of endothelin-1, in particular EPA supplementation in human endothelial cells determined lower levels of this potent vasoconstrictor, independently of the production of NO [108]. Evidence from a human study demonstrated that a 12-week intervention with EPA and DHA in older subjects was able to decrease carotid-femoral PWV, an important measure of arterial stiffness, but not arterial blood pressure and arterial wave reflections (central augmentation index) [109].

On the other hand, the role of ω-6 FA on vascular function is still controversial and dependent on the individual FA considered. Despite the possible rationale to the effect of linoleic acid (LA) in the amelioration of vascular function, it should be underlined that evidence of the effects of specific FA in vascular function, even they are not conclusive and overall limited, currently leans more towards an effect of ω-3 PUFA on vascular function, rather than ω-6 PUFA [103]. Dietary essential fatty acids (EFA) are modified by specific enzymes to produce the eicosanoids, i.e., second messengers highly produced by endothelial cells and particularly relevant for CV health [110]. For instance, from arachidonic acid (AA), a well-known metabolite of LA, derives prostacyclin (PGI_2_), an important contributor to vasodilation together with NO [110], but also thromboxane A2 (TXA2), a well-known vasoconstrictor factor [111]. Furthermore, Hennig et al. also reported that LA hydroperoxide may be associated with endothelial dysfunction and atherogenesis through inflammation-promoting effects [112]. Moreover, several mechanisms through which LA exerts its negative role on vascular functions are supposed. Among them, LA mainly consumed from refined vegetable oils is incorporated in low density lipoproteins (LDL) and seems to be responsible for triggering LDL oxidation due its high susceptibility to oxidative processes. Once oxidized, apoB LDL is not recognized by liver receptor (due to its binding with oxidation-derived products such as malondialdehyde and 4-hydroxynonenal), while macrophages, through their scavenger receptor, are able to internalize ox-LDL and promote atherosclerotic plaque development [113]. Additionally, lipoprotein lipase at the level of the endothelium acts on very low-density lipoproteins (VLDL) determining the release of oxidized lipids from linoleic acid such as 13-hydroxyoctadecadienoic acid. These metabolites represent a harmful stimulus directly to the endothelium, by enhancing the release of ROS (mainly by inducing p47 and NADPH oxidase enzyme complex) and cell adhesion molecules (CAMs) and exacerbating the inflammatory process and vascular permeability [113]. However, a systematic review that specifically addressed this hypothesis found that no evidence from randomized, controlled intervention studies on healthy subjects indicates a negative effect of LA on inflammation markers [114]. Furthermore, a systematic review highlighted that changes in LA intake are not associated with significant variation in tissue AA content in an adult population consuming a Western-type diet [115]. Thus, another possible interpretation of the results indicated by de Oliveira P. et al. [70] that should be considered is a matrix effect in which other bioactives (e.g., sunflower oil polyphenols) contributed to the amelioration of vascular response [116].

In contrast to ω-6 PUFA, ω-9 MUFA are less susceptible to oxidation, indeed, in samples of human atheroma the amount of oxidized LA was higher than oxidized oleic acid (OA) [117]. In Sprague Dawley rat cardiomyocytes, OA reduced oxidative stress and inflammation induced by SFA, exerting an antiatherogenic effect [118]. Additionally, in healthy individuals, the enrichment of milk with oleic acid was able to lower total cholesterol (TC), low-density lipoproteins cholesterol (LDL-C), and triglycerides (TG) levels [119]. Different clinical studies suggest a beneficial effect of OA, the main constituent of olive oil, on markers of endothelial function such as FMD [102]. At the same time, some studies using in vitro models reported a controversial role of OA on vascular function. For instance, it has been documented that OA induced the disruption of calcium signaling and the following NO production and release in aortic endothelial cells [120].

Besides unsaturated FA, the detrimental role on vascular function attributed to SFA is due to their effect on increasing TC and LDL-C, both markers of CV risk. However, results are still being debated and, again, depend on the individual SFA and also the triacylglycerol structure that could determine a potential different effect [121]. In an RCT conducted on 195 subjects with moderate CVD risk, the replacement of SFA with MUFA or ω-6 PUFA did not observe effect on vascular function measured as FMD and other measures of arterial stiffness such as AI and PWV, while the same intervention determined a reduction of night systolic blood pressure, E-selectin, TC, and LDL-C [122]. Although a short-term intervention, healthy subjects supplemented with a diet rich in SFA observed a reduction in FMD and increased P-selectin levels compared to MUFA and PUFA groups [123]. Conversely, another RCT did not observe differences between MUFA- or SFA-based oils in endothelium-dependent and independent vascular responses [124]. Results deriving from in vitro studies indicate a detrimental effect of SFA on endothelial function through mitogen-activated protein kinase (MAPK) pathway that leads to endothelin 1 (ET-1) induction and the following vasoconstriction, but also an increase in CAMs and plasminogen activator inhibitor-1 (PAI-1) [125].

Taken together, the existing evidence suggests a key role of vegetable oils and their relative main FAs on vascular function, and also several potential mechanisms of action have been assumed. However, the scarcity of well-designed interventional studies does not allow a clear interpretation of the role of individual vegetable oils/fatty acids.

## 5. Strengths and Limitations

Overall, this review shows several strengths and limitations. The main strength regards the systematic approach used for the searching and the selection of the studies. In fact, the use of different databases allowed us to be more inclusive in our research. In addition, the choice to focus only on older subjects (aged more than 60 years), by excluding studies including mixed populations, but not only older individuals, has allowed us to have a clear overview of the current studies available on this target population. Another important strength is the assessment of the study quality by using validated tools able to provide detailed information on the methodological quality (risk of bias) of the studies included and to make a weight of the evidence. Regarding the limitations, the first one is related to the few dietary intervention trials available in older subjects. The second is related to the high heterogeneity among studies in terms of the type and dose of products tested, way of administration (i.e., fruit/product, drink), nutritional composition/characterization, and also in terms of bioactives. Third, differences in terms of experimental protocols adopted (i.e., acute versus medium-long term, with and/or without physical activity) and characteristics of the study population (i.e., healthy and/or with risk factors) make the comparison among studies difficult to perform. Lastly, differences in terms of outcomes (i.e., FMD, PWV, AI) and their measurements (i.e., gold standard techniques versus surrogate methodologies). All these limitations made it impossible to perform a quantitative analysis (meta-analysis) of the effects of the different plant-based products tested.

## 6. Conclusions and Future Directions

In conclusion, the results of the studies included in the present review, although few and with several limitations, seem to corroborate the possible contribution of selected plant-based foods in the improvement of vascular function in older subjects. In particular, the main effects seem to derive from beetroots and their bioactives (such as nitrate/nitrite and betalains) on markers of vascular reactivity including FMD, FBF, and BFV, while no changes were found for markers of arterial stiffness (i.e., AI, PWV). The effects on vascular function seem to be more related to the restoration of a condition of nitric oxide impairment (exacerbated by diseases or hypoxic condition), which in turn ameliorates vascular function, rather than the enhancement of a normal physiological condition. Interestingly, the positive observation seems also mediated by a contribution of oral microbiota able to reduce NO3- into NO2- and in turn exert a physiological effect. However, the limitations of the studies described above, mainly in terms of heterogeneity among trials, reduce the significance of the findings on beetroots. This aspect also regards the results of the studies performed by using plums, blueberries, and vegetable oils that are too limited. Thus, future well-designed intervention studies focused on this target group are highly recommended to corroborate the current findings and to better elucidate the mechanisms through which plant-based foods may help in improving vascular health in aging. In addition, dose and time-dependent studies are needed to better identify the products, and related bioactives which are able to have a vasodilator effect. The results of such studies could be useful for the development of new products able to maintain and/or improve vascular health and reduce the incidence of vascular disorders in older subjects.

## Figures and Tables

**Table 1:** Characteristics of the acute intervention studies investigating the effects of plant-food consumption on markers of vascular function in older subjects.

Reference, Country	Study Design	Study Population	Test Product	Control Product	Outcome Variables	Main Findings
Casey et al. 2015, USA [58]	Randomized, parallel, placebo-controlled	*n* = 12 healthy older subjects (9 M + 3 F), of which 7 completed the placebo trial Mean age = 64 ± 2 years Mean BMI = 25.5 ± 0.7 kg/m^2^	500 mL of beetroot juice providing 9.4 mmol of nitrate	140 mL of nitrate-deprived beetroot juice + 360 mL of water	FBF, FVC, and CV tested in both condition of normoxia and hypoxia, at rest and after forearm exercise Tested prior and after 3 h from consumption of test product or placebo	↑ FBF, and FVC and in hypoxia compared to control in older subjects, but not in young subjects ⟷ FBF and FVC observed in the placebo group under both normoxic and hypoxic condition
de Oliveira et al. 2016, Brazil [56]	Randomized, crossover, placebo-controlled, double-blind Washout period of at least 1 week	*n* = 20 older subjects with cardiovascular risk factors (7 M + 13 F). Mean age = 70.5 ± 5.6 years Mean BMI = 30.2 ± 5.3 kg/m^2^	100 g of beetroot gel providing 12.2 mmol of nitrate and 367.9 mg of phenolic acids (expressed as GAE)	100 g of nitrate-deprived beetroot gel (placebo)	FMD, RH, BFV, PWV, AIx, Ep, and AC. Tested 2 h after consumption of the test or control product	↑ FMD, RH, and BFV in test products vs. placebo ⟷ arterial stiffness parameters
Dodd et al. 2019, UK [61]	Randomized, crossover, controlled, double blind Washout period not indicated	*n* = 18 older subjects not taking anti-hypertensive medications (8 M + 13 F)Mean age = 68.7 ± 3.3 years Mean BMI = 25.9 ± 4.5 kg/m^2^	30 g of blueberry powder providing about 500 mg of anthocyanidins and 70 mg of procyanidins, homogenized with 300 mL of semi-skimmed milk and consumed after a standardized breakfast	30 g of a powder providing the same amount of sugar and vit. C of blueberry powder.	DVP Tested 1 h after consumption of the test or control product	⟷ arterial stiffness parameters
Hughes et al. 2016, USA [57]	Comparative parallel study evaluating the differences in response to acute ingestion of dietary nitrate between young and older subjects. Uncontrolled for older subject group.	*n* = 12 healthy non-obese, non-hypertensive older subjects (9 M + 3 F). Mean age = 64 ± 5 years Mean BMI = 25.5 ± 2 kg/m^2^	500 mL of beetroot juice providing 9.4 mmol of nitrate	-	AIx, AIx@75, and other hemodynamic measures Tested at six different timepoints (baseline, 1 h, 1,5 h, 2 h, 2,5 h, and 3 h).	⟷ arterial stiffness and other parameters
Hughes et al. 2020, USA [59]	Randomized, crossover, placebo-controlled, double-blind	*n* = 10 healthy non-obese non-smokers older subjects (7 M + 3 F). Mean age = 68 ± 1 years Mean BMI = 25.8 ± 1 kg/m^2^	Beetroot powder in 240 mL of water, providing 4 mmol of nitrates and 0.3 mmol of nitrites.	Nitrate-deprived beetroot powder in 240 mL of water.	Leg BF and VC, tested during exercise Tested prior and 2 h after consumption of test product or placebo	⟷ vascular reactivity parameters
Pekas et al. 2021 USA [60]	Randomized, crossover, placebo-controlled, double-blind Washout period of 14 days	*n* = 11 older subjects with PAD (5 M + 6 F). Mean age = 70 ± 7 years Mean BMI = 29 ± 6 kg/m^2^	Body mass-normalized beetroot juice, providing 0.11 mmol of nitrates/kg	Nitrate deprived beetroot beverage	Brachial and popliteal FMD, AIx, AI@75, AP, PP, PWV Tested prior and 1 h after consumption of test product or placebo	↑ FMD in test products vs. placebo

Abbreviations: AC, arterial compliance; AIx, augmentation index; AIx@75, AIx normalized by considering a heart rate of 75 bpm; AP, augmented pressure; BMI, body mass index; BF, blood flow; BFV, blood flow velocity; CV, compensatory vasodilation; DVP, digital volume pulse; Ep, pressure-strain elasticity modulus; FBF, forearm blood flow; FMD, flow-mediated dilation; FVC, forearm vascular conductance; GAE, gallic acid equivalents; pulse wave velocity; PAD, Peripheral Artery Disease; PP, pulse pressure; PWV, carotid-to-femoral pulse-wave velocity; RH, reactive hyperemia; VC, vascular conductance.

**Table 2:** Characteristics of the chronic intervention studies investigating the effects of plant-food consumption on markers of vascular function in older subjects.

Reference, Country	Study Design	Study Population	Test Product	Control Product	Duration	Outcome Variables	Main Findings
Casey and Bock. 2021, USA [62]	Randomized, cross-over, placebo-controlled, double-blind Washout period of at least 4 weeks	*n* = 10 healthy, non-obese, non-smokers older subjects (6 M + 4 F) Mean age = 67 ± 3 years Mean BMI >25.8 ± 3.3 kg/m^2^.	Beetroot powder mixed with approximately 200 mL of water, providing about 4 mmol of nitrate and 0.3 mmol of nitrate	Nitrate- and nitrite-depleted Beetroot powder	4 weeks	Measures of shear profile at rest, and measure of exercise hyperemia during handgrip exercise (FBF and FVC).	Improvement in shear profile in test products group but not in placebo group
de Oliveira et al. 2017, Brazil [70]	Randomized, parallel, double-blind	*n* = 76 obese or overweight non-diabetic older subjects (23 M + 53 F) Mean age = 67.4 ± 5.16 years Mean BMI >28 kg/m^2^	30 mL/day of three different types of vegetable oil: olive, flaxseed or sunflower oil. This quantity of oil should not have been additional to the normal diet of the subjects	Absent	90 days	FMD and CIMT	↑ FMD in the group that consumed sunflower oil ↓ CIMT in all the intervention groups
do Rosario et al. 2020, Australia [64]	Randomized, crossover, placebo-controlled, double-blind Washout period of 14 days	*n* = 16 overweight or obese, but otherwise clinically healthy elderly subjects (non-hypertensive, non-diabetic, non-drug-treated, non-smoker subjects). Mean age = 65.9 ± 6 years Mean BMI = 30.6 ± 3.9 kg/m^2^	250 mL/day of high ACN queen garnet plum juice (about 200 mg of ACN)/day	250 mL colored apricot juice (placebo).	5 days	FMD and microvascular reactivity (peak shear rate, PV, PORH max, and IONT max) to evaluate postprandial response 2 h after challenging with a high energy/high fat test meal	↑ FMD, and PORH max in test products group versus control
do Rosario et al. 2020, Australia [63]	Randomized, parallel, placebo-controlled, double-blind	*n* = 31 older subjects with mild cognitive impairment (12 M + 19 F). Mean age = 75.3 ± 6.9 years Mean BMI = 26.1 ± 3.3 kg/m^2^	250 mL/day of two different types of fruit juice: low ACN queen garnet plum (about 45 mg of ACN) or high ACN queen garnet plum (about 200 mg of ACN).	Colored apricot juice (placebo). Blinding strategies included advertising and consenting participants to a “fruit juice study”, without providing information on which fruit was being investigated.	8 weeks	Microvascular reactivity evaluated POHR	⟷ microvascular reactivity
Gilchrist et al. 2013, UK [65]	Randomized, crossover, placebo-controlled, double-blind Washout period of 4 weeks	*n* = 27 non-smokers older subjects with T2DM (of at least 5 years duration) and BP 4125/85 mm Hg or on one or more antihypertensive agents (18 M + 9 F). Mean age = 67.2 ± 4.9 years. Mean BMI = 30.8 ± 3.2 kg/m^2^	250 mL/day of beetroot juice, providing 7.5 mmol of nitrate	250 mL of nitrate-depleted beetroot juice (placebo).	2 weeks	FMD and microvascular endothelial function (perfusion response after skin iontophoresis of ACh and SNP)	⟷ macro- and microvascular reactivity
Jones et al. 2019, UK [69]	Randomized, parallel, placebo-controlled, double-blind	*n* = 20 non-obese older subjects. Mean age = 63 ± 6 years. Mean BMI = 26,5 kg/m^2^	70 mL of beetroot juice providing 4.7 mmol of nitrate every day	70 mL of prune juice	4 weeks	FMD and markers of microvascular endothelial function (perfusion response after skin iontophoresis of ACh and SNP)	⟷ compared to placebo
Oggioni et al. 2017, UK [66]	Randomized, crossover, placebo-controlled, double-blind Washout period of at least 7 days	*n* = 20 non-smokers healthy older subjects (10 M + 10 F). Mean age = 64.7 ± 3 years. Mean BMI = 25.6 ± 3.4 kg/m^2^.	70 mL of beetroot juice twice a day (for a total of 12 mmol additional nitrate/day)	70 mL of nitrate-depleted beetroot juice twice a day (placebo)	1 week	AIx	⟷ arterial stiffness parameters
Shaltout et al. 2017, USA [67]	Randomized, parallel, placebo-controlled, double-blind	26 older hypertensive patients (13 M + 13 F). Mean age = 69 ± 7 years. Mean BMI = 33.7 kg/m^2^.	70 mL of beetroot juice providing 6.1 mmol of nitrate every day + aerobic exercise training 3 times/week	70 mL of nitrate-depleted beetroot juice twice a day (placebo) + aerobic exercise training	6 weeks	Hemodynamic measures (SVR, TAC, VI, AI, and LCWI)	⟷ hemodynamic measures
Woessner et al. 2018, USA [68]	Randomized, parallel, placebo-controlled, double-blind	*n* = 24 older subjects with PAD + IC (10 M + 10 F). Mean age = 64.7 ± 3 years. Mean BMI = 25.6 ± 3.4 kg/m^2^.	70 mL of beetroot juice providing 4.2 mmol 3 h prior training session (30 min walking sessions 3 times/week)	70 mL of nitrate-depleted beet-root juice twice a day (placebo) prior training session	12 weeks	ABI and RHBF	↑ ABI, and RHBF in the test group vs. placebo

Abbreviations: ABI, resting ankle-brachial index; Ach and SNP, acetylcholine and sodium nitroprusside; ACN, anthocyanin; AI, acceleration index; AIx, augmentation index; BMI, body mass index; CIMT, carotid intima-media thickness; D, brachial artery diameter; FBF, forearm blood flow; FMD, flow-mediated dilation; FVC, forearm vascular conductance; IONT max, maximum perfusion following iontophoresis of acetylcholine; LCWI, left cardiac work index; LSCI combined with POHR, laser speckle contrast imaging combined with a post-occlusive reactive hyperemia test; PAD + IC, peripheral artery disease associated with intermittent claudication; PORH max, post-occlusive reactive hyperemia maximum perfusion; PV, peak value; RHBF, reactive hyperemic blood flow; SVR, systemic vascular resistance; TAC, total arterial compliance; T2DM, type 2 diabetes mellitus; VI, velocity index; V_mean_, blood velocity; n.r., not reported.

## Data Availability

Not applicable.

## Supplementary Materials

The following are available online at https://www.mdpi.com/article/10.3390/nu14132615/s1, Figure S1: Risk of bias for each item assessed in each of the included studies; Figure S2: Risk of bias for each item assessed, presented as a percentage across all included studies combined.
