# Supplementary material for: Plant-Based Foods and Vascular Function: A Systematic Review of Dietary Intervention Trials in Older Subjects and Hypothesized Mechanisms of Action
Source: Nutrients. 2022 Jun 24;14(13):2615. doi: 10.3390/nu14132615 (PMC9268664; doi:10.3390/nu14132615)

Supplementary Materials

Figure S1. Risk of bias for each item assessed in each of the included studies.

	(1) random sequence generation	(2) allocation concealment	(3) blinding of participants and personnel	(4) blinding of outcome assessment	(5) incomplete outcome data	(6) selective reporting data	(7) other bias
Casey et al., 2015	?	?	?	-	+	-	?
Dodd et al., 2019	?	?	+	?	+	-	?
Hughes et al., 2016	?	?	?	-	-	?	?
Hughes et al., 2020	?	?	+	?	+	?	?
Pekas et al., 2021	?	?	+	+	+	-	?
Viera de Oliveira et al., 2016	?	?	+	+	+	?	?
Amante de Oliveira et al., 2017	?	?	+	+	+	?	?
Casey & Bock 2021	?	?	+	?	+	?	?
do Rosario et al., 2020	+	+	+	?	+	?	?
Gilchrist et al. 2013	?	?	+	?	+	?	?
Jones et al. 2019	-	-	+	+	+	?	?
Oggioni et al., 2017	?	?	+	?	+	?	?
Shaltout et al., 2017	+	?	+	+	+	?	?
Woessner et al., 2018	?	?	+	?	+	?	?
do Rosario et al., 2020	+	+	+	+	+	?	?

Figure S2. Risk of bias for each item assessed, presented as a percentage across all included studies combined.

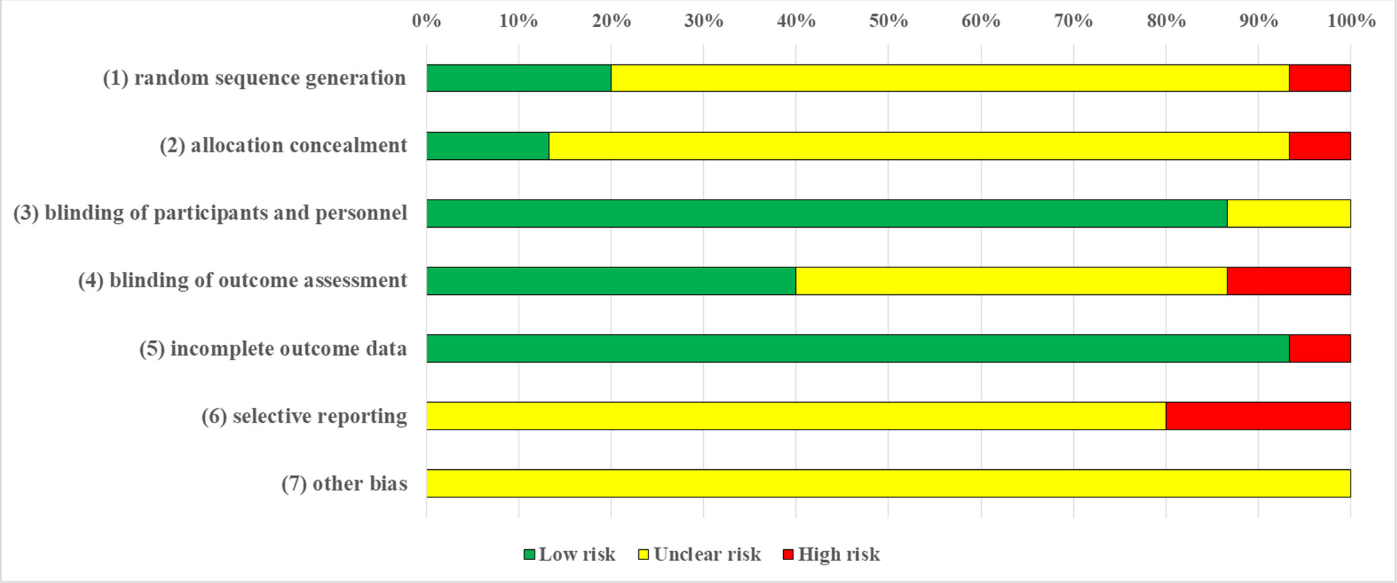

Supplement: Supplementary file 1 [file nutrients-14-02615-s001.zip › nutrients-1772386-supplementary.pdf]
